# Functional Characterization of *GA2ox3* in Rice Osmotic Stress Response and Identification of a Superior Allele for Breeding Applications

**DOI:** 10.3390/plants15081205

**Published:** 2026-04-15

**Authors:** Liping Dai, Xujie Chen, Danni Yao, Huihui Sun, Qianwen Li, Ting Yang, Jinwei Qi, Chengyi Zhang, Xinyao Li, Kehui Zhang, Hui Wang, Chaohuang Tu, Yujue Wang, Chengfang Zhan, Xueli Lu, Guanghui Xiao, Dali Zeng

**Affiliations:** College of Advanced Agricultural Sciences, Zhejiang A&F University, Hangzhou 311300, China; dailiping777@126.com (L.D.); 18368866206@163.com (X.C.); ydn6868668@163.com (D.Y.); 13115725697@163.com (H.S.); liqianwen2026@126.com (Q.L.); m18857914218@163.com (T.Y.); qijinweihainan@163.com (J.Q.); 19157737041@163.com (C.Z.); 18636188645@163.com (X.L.); m19730567903@163.com (K.Z.); 19855918187@163.com (H.W.); lingting241@163.com (C.T.); 19738640108@163.com (Y.W.); zhanchf@zafu.edu.cn (C.Z.); luxueli6666@163.com (X.L.); guanghuix1214@gmail.com (G.X.)

**Keywords:** rice (*Oryza sativa* L.), *GA2ox3*, drought tolerance, haplotype analysis, nature variation

## Abstract

Drought is a major abiotic stress limiting rice production worldwide. *GA2ox3*, a GA 2-oxidase involved in gibberellin deactivation, has been previously linked to growth regulation. Here, we demonstrate that functional disruption of *GA2ox3* enhances shoot growth but compromises drought tolerance in rice. CRISPR/Cas9 knockout lines (*ga2ox3*) showed increased plant height and reduced antioxidant capacity under 20%PEG-induced osmotic stress, evidenced by higher H_2_O_2_ and MDA accumulation and decreased POD activity. Moreover, expression analysis revealed that *GA2ox3* may be associated with the GA signaling cascade involving *OsSLR1*, *OsBURP3*, and *OsSUS1*. Haplotype analysis based on 627 rice accessions identified two major alleles, Hap1 and Hap2, with Hap1 showing higher *GA2ox3* expression and stronger drought resilience. These findings suggest that *GA2ox3* positively regulates osmotic stress tolerance, and its natural variation represents promising genetic resources for improving drought adaptation in rice.

## 1. Introduction

Rice (*Oryza sativa* L.) is one of the most important staple crops worldwide, serving as the primary food source for more than half of the global population [1]. Drought, one of the most severe abiotic stresses, causes substantial yield losses globally each year and severely limits crop productivity and sustainability. Therefore, identifying key genes involved in drought tolerance has become a major focus in crop genetic improvement and molecular breeding.

Gibberellins (GAs) are important phytohormones that widely regulate various developmental processes, including seed germination, stem elongation, and flowering, as well as adaptive responses to abiotic stresses such as drought, salinity, and cold [1]. Numerous studies have shown that GA signaling plays a critical role in plant responses to drought stress [2]. For instance, in *Arabidopsis*, the DELLA protein GAI interacts with the transcription factor ABF2 to positively regulate the expression of drought-tolerant genes [3]; in rice, *OsSLR1* (encoding a DELLA protein) enhances drought tolerance by regulating the *OsBURP3*-*OsSUS1* module, thereby reducing the nuclear accumulation of *OsSUS1* [4]. In addition, GA signaling participates in drought adaptation through multiple physiological pathways, including the regulation of stomatal aperture, accumulation of osmotic adjustment substances, and antioxidant systems [5]. Collectively, these findings indicate a close association between GA signaling and drought stress responses.

The bioactive GA levels in plants are tightly controlled by the balance between biosynthesis and deactivation. Among the enzymes involved, GA 2-oxidases (GA2ox) catalyze the inactivation of bioactive GAs and are key regulators of GA homeostasis [6]. Recent studies have shown that *GA2ox* family members also play important roles in plant responses to abiotic stress, but their functions exhibit diversity across different species and stress conditions. ArabidopsisGA2ox is involved in responses to salt and cold stress: under high salinity, the transcription factor DDF1 promotes *AtGA2ox7* expression, resulting in growth inhibition that helps Arabidopsis adapt to salt stress [7]; similarly, *AtGA2ox9* expression is significantly upregulated following cold treatment, contributing to cold tolerance [8]. Similarly, in cotton, overexpression of *GhGA2ox1*enhances tolerance to both drought and salt stress [9]. Notably, even within the same species (maize), different *GA2ox* family members exhibit clear functional divergence: most GA2oxgenes show distinct expression patterns under various abiotic stresses, and even under the same stress condition, the expression of different family members can be completely opposite-for example, under salt stress, *ZmGA2ox6* and *ZmGA2ox9* are upregulated while *ZmGA2ox5* is downregulated; under drought stress, *ZmGA2ox1;1* is upregulated while *ZmGA2ox1;2* is downregulated; moreover, some *GA2ox* genes show no significant change in expression under stress conditions [10]. These findings collectively indicate that *GA2ox* family members exhibit significant functional divergence in response to different abiotic stresses, and their modes of action vary depending on species, stress type, and family member. However, *GA2ox3*, one of the better-characterized members of the *GA2ox* family in rice [11,12,13,14], encodes a GA-deactivating enzyme known to suppress plant height [15], and its role in ethylene-regulated root development has been reported. However, these studies have mainly focused on its functions in growth and development, while its role in response to osmotic stress remains unclear. Therefore, in this study, we selected *GA2ox3* as a candidate gene to investigate its function in osmotic stress tolerance in rice.

In this study, we generated *GA2ox3* knockout mutants and performed phenotypic, physiological, and expression analyses under PEG-induced osmotic stress to investigate the role of this gene in response to osmotic stress in rice. In addition, we conducted haplotype analysis using whole-genome resequencing data from 627 rice accessions to identify superior allelic variations associated with drought tolerance.

## 2. Results

### 2.1. Loss of GA2ox3 Function Enhances Shoot Growth in Rice Seedlings

**Figure 1:**
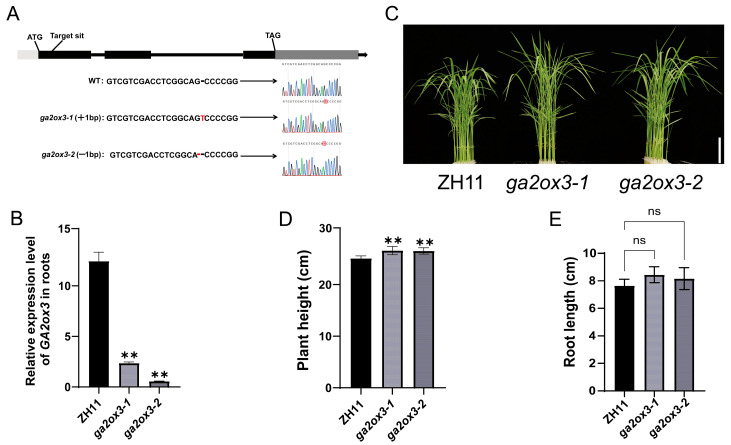
Phenotypic characterization of *ga2ox3* in rice seedlings. (**A**) Schematic diagram of *GA2ox3* structure and edited homozygous transgenic rice lines at *GA2ox3* locus by CRISPR/Cas9. White rectangles represent the 5′ UTR, black rectangles represent the exons, black lines represent the introns, gray rectangles represent the 3′ UTR, and red text or lines represent the mutation sites. F1 and R1 indicate the positions of the primers used for gene expression analysis. (**B**) Relative expression levels of *GA2ox3* in ZH11 and *ga2ox3* shoots determined by qRT-PCR. (**C**) Phenotypes of ZH11 and *ga2ox3* seedlings grown under normal conditions for 10 days. Bar = 5 cm. (**D**) Comparison of the shoot length between ZH11 and *ga2ox3*. (**E**) Comparison of primary root length between ZH11 and *ga2ox3*; Values are presented as mean ± SD from five biological replicates. Statistical significance was determined by Student’s *t*-test (** *p* < 0.01; ns indicates no significant difference).

The gene structure of *GA2ox3* was analyzed using the Rice Genome Annotation Project database. The results showed that *GA2ox3* is located on chromosome 1 and contains three exons and two introns, with a coding sequence of 984 bp (Figure 1A). To investigate the function of *GA2ox3*, particularly its role in response to osmotic stress, we generated a CRISPR/Cas9-based gene knockout vector targeting *GA2ox3* and obtained regenerated plants via Agrobacterium-mediated transformation. Two independent T0 homozygous lines were selected by Sanger sequencing and designated as *ga2ox3-1* and *ga2ox3-2*. Among them, *ga2ox3-1* carries a single “T” nucleotide insertion between the 252nd and 253rd bases downstream of the start codon, while *ga2ox3-2* has a deletion of a “G” nucleotide at the 252nd base, both resulting in frameshift mutations that introduce premature stop codons (TGA) in exon 3 (amino acid position 267) and exon 2 (amino acid position 133), respectively. qRT-PCR analysis showed that the expression level of *GA2ox3* was significantly downregulated in both mutants compared to ZH11 (Figure 1B). Meanwhile, phenotypic analysis revealed that both *ga2ox3-1* and *ga2ox3-2* exhibited significantly increased plant height compared to the wild type (Figure 1C).

Phenotypic observations and statistical analysis of 10-day-old seedlings revealed that the shoot length of *ga2ox3* mutants was approximately 26.6 cm, which was highly significantly taller than that of wild-type ZH11 at approximately 25.1 cm (Figure 1D). However, there was no significant difference in root length between *ga2ox3* (8.3 cm) and ZH11 (7.6 cm) (Figure 1E). These results suggest that loss of *GA2ox3* function promotes shoot growth in rice.

### 2.2. Disruption of GA2ox3 Increases Plant Height Without Affecting Yield-Related Traits

**Figure 2:**
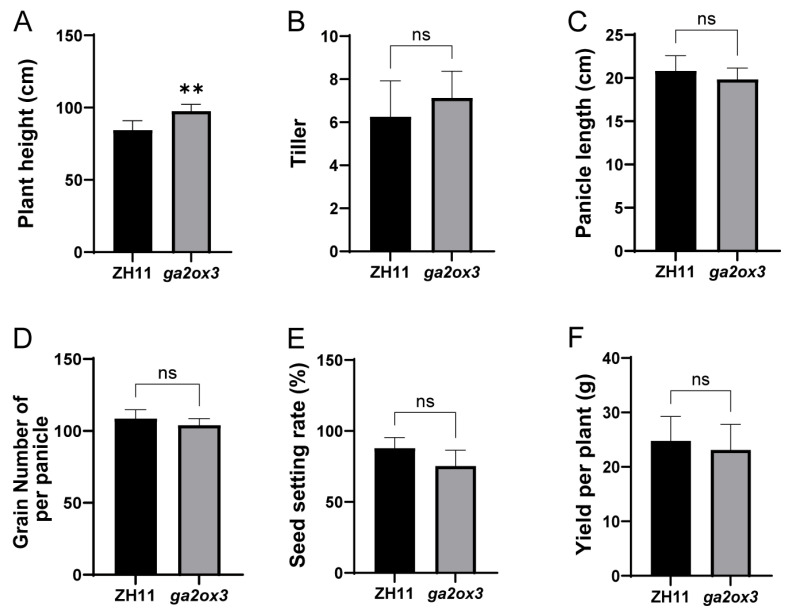
Agronomic trait comparison between ZH11 and *ga2ox3* at maturity stage. (**A**) Plant height at maturity stage; (**B**) Tiller number; (**C**) Panicle length; (**D**) Number of grains per panicle; (**E**) Seed setting rate; (**F**) Grain yield per plant. Values are presented as mean ± SD (*n* = 8 for plant height and tiller number; *n* = 4 for panicle length, grains per panicle, seed setting rate, and grain yield per plant). Statistical significance was determined by Student’s *t*-test (** *p* < 0.01; ns indicates no significant difference).

To determine whether loss of *GA2ox3* function affects rice yield, we conducted phenotypic analysis of ZH11 and *ga2ox3* plants. The results showed that the plant height of *ga2ox3* was 97.60 cm, representing a 15.6% increase compared to ZH11 (Figure 2A). However, the *ga2ox3* showed no significant differences from ZH11 in yield-related traits, including tiller number (7.1), panicle length (19.9 cm), number of grains per panicle (104), seed setting rate (75.32%), and grain yield per plant (23.1 g) (Figure 2B–F). These results indicate that the loss of *GA2ox3* function specifically promotes shoot elongation without negatively affecting tillering, fertility, or yield components in rice.

### 2.3. Spatiotemporal Expression Pattern of GA2ox3

**Figure 3:**
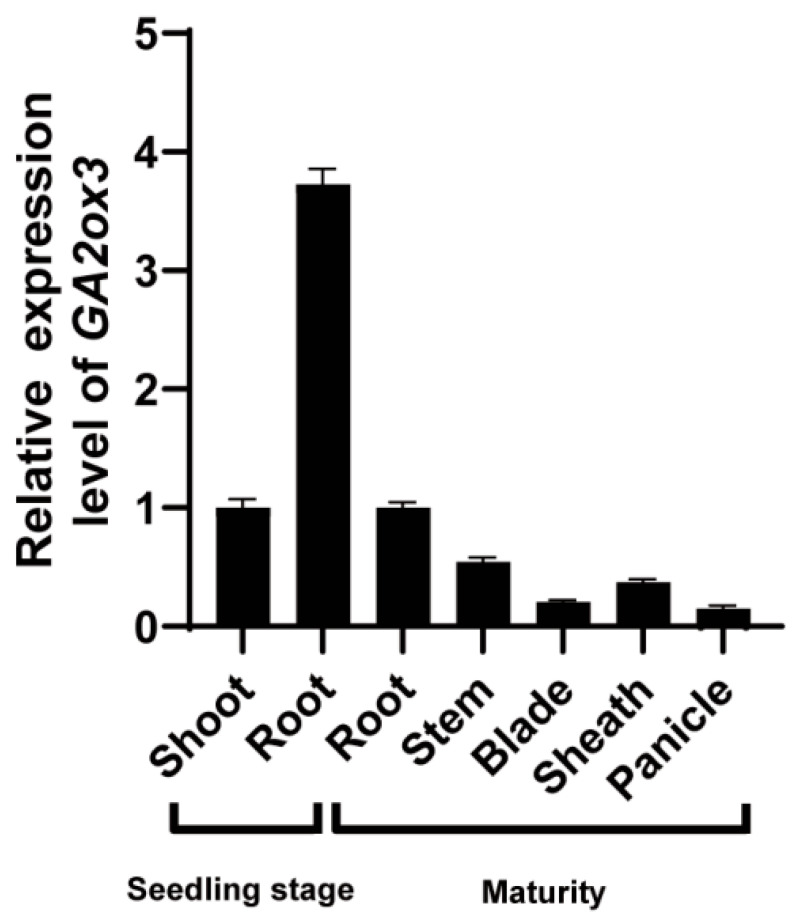
Spatiotemporal expression pattern of *ga2ox3*. Relative expression levels of *GA2ox3* in different tissues at seedling stage (shoot and root) and heading stage (root, stem, leaf blade, sheath, and panicle). All samples were collected from Nipponbare. Values are presented as mean ± SD (*n* = 3 biological replicates).

To investigate the spatiotemporal expression pattern of *GA2ox3* under normal growth conditions, total RNA was extracted from various tissues and developmental stages of rice plants for qRT-PCR analysis. The results showed that *GA2ox3* was transcribed in all examined tissues, including shoot and root at the seedling stage, as well as root, stem, leaf blade, sheath, and panicle at the heading stage. Notably, its transcript level was highest in roots at the seedling stage, followed by seedling shoots and roots at the heading stage (Figure 3).

### 2.4. Functional Loss of GA2ox3 Increases Sensitivity to Osmotic Stress

Osmotic stress is one of the main factors contributing to plant damage under osmotic stress. To assess the role of *GA2ox3* in response to osmotic stress, 10-day-old seedlings of wild-type ZH11 and *ga2ox3* were subjected to either 0% (control) or 20% polyethylene glycol (PEG) treatment for 14 days, followed by phenotypic observation and statistical analysis.

**Figure 4:**
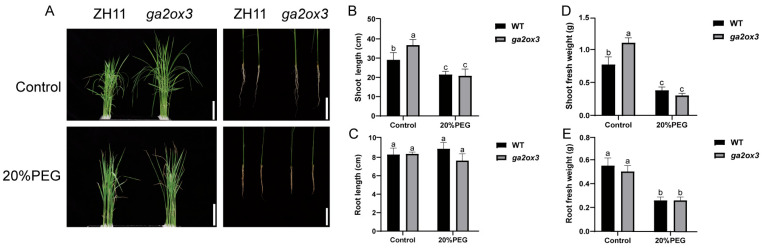
Phenotypic characterization of ZH11 and *ga2ox3* under normal and PEG-induced osmotic stress conditions. (**A**) The phenotypic characteristics of ZH11 and *ga2ox3* seedlings after 14 days of treatment with normal conditions and 20% PEG. Bar = 5 cm. (**B**) The plant heights, (**C**) root lengths, (**D**) shoot fresh weight and (**E**) root fresh weight of the ZH11 and *ga2ox3* seedlings under normal conditions and 20% PEG treatment. Values are presented as mean ± SD. *p*-values were calculated using ANOVA. The same letters indicate no significant difference between groups (*p* > 0.05), while different letters denote statistical significance (*p* < 0.05).

Under control conditions, all lines exhibited normal growth, with average shoot lengths of 29.12 cm in ZH11 and 36.75 cm in *ga2ox3*. Under 20% PEG treatment, all plants developed tip burn, a symptom that was markedly more severe in the *ga2ox3* mutant. Consistently, shoot lengths decreased to 21.43 cm in ZH11 and 20.81 cm in *ga2ox3*, representing reductions of 26.41% and 43.37%, respectively (Figure 4A,B). While shoot length was significantly affected by PEG treatment, no significant change in root length was observed in either genotype under control or PEG-treated conditions (Figure 4C). Under stress conditions, the fresh weight of both shoot and root tissues was significantly reduced in both genotypes compared to their respective controls, and this reduction was more pronounced in *ga2ox3* (Figure 4D,E).

**Figure 5:**
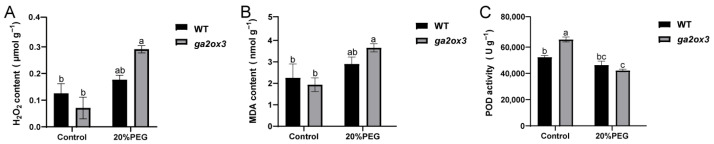
Determination of ROS content in the ZH11 and *ga2ox3* under normal conditions and 20% PEG conditions. (**A**) H_2_O_2_ content, (**B**) MDA content, and (**C**) POD activity of the shoots of ZH11 and *ga2ox3* seedlings under normal conditions and 20% PEG treatment. Values are presented as mean ± SD from three biological replicates. *p*-values were calculated using ANOVA. The same letters indicate no significant difference between groups (*p* > 0.05), while different letters denote statistical significance (*p* < 0.05).

To investigate whether *GA2ox3* affects the accumulation and scavenging of reactive oxygen species (ROS), we measured the content of hydrogen peroxide (H_2_O_2_) and malondialdehyde (MDA), as well as the activity of peroxidase (POD) in both control and 20%PEG-treated plants. Under PEG treatment, H_2_O_2_ content in ZH11 showed a slight increase compared to the control, which was not statistically significant, whereas in *ga2ox3* it increased sharply by 3.14-fold, a highly significant difference (Figure 5A). In addition, we measured malondialdehyde (MDA) content which is the final product of membrane lipid peroxidation and a key indicator of oxidative damage. The 20% PEG treatment caused no significant change in MDA content in ZH11, whereas MDA content in *ga2ox3* increased significantly by approximately 2-fold, indicating severe membrane lipid peroxidation damage in the mutant (Figure 5B). Furthermore, POD activity, a key ROS-scavenging enzyme, was significantly reduced in both 20%PEG-treated ZH11 and *ga2ox3*, reaching 88.56% and 64.12% of their respective controls (Figure 5C). These findings demonstrate that the loss of *GA2ox3* function compromises tolerance to osmotic stress in rice, likely due to impaired antioxidant defenses and excessive ROS accumulation.

### 2.5. Altered Expression of SLR1, BURP3, and SUS1 in Response to Osmotic Stress in GA2ox3 Knockout Lines

Previous studies have shown that *OsSLR1*, a DELLA protein, negatively regulates the expression of the dehydration-responsive gene *OsBURP3*, which in turn reduces the nuclear accumulation of *OsSUS1*, ultimately enhancing drought tolerance in rice [2]. To further explore the regulatory network involving *GA2ox3*, we examined the expression of three key genes in the stress pathway-*OsSLR1*, *OsBURP3*, and *OsSUS1*-in both ZH11 and *ga2ox3* lines under control and 20% PEG treatment conditions.

**Figure 6:**
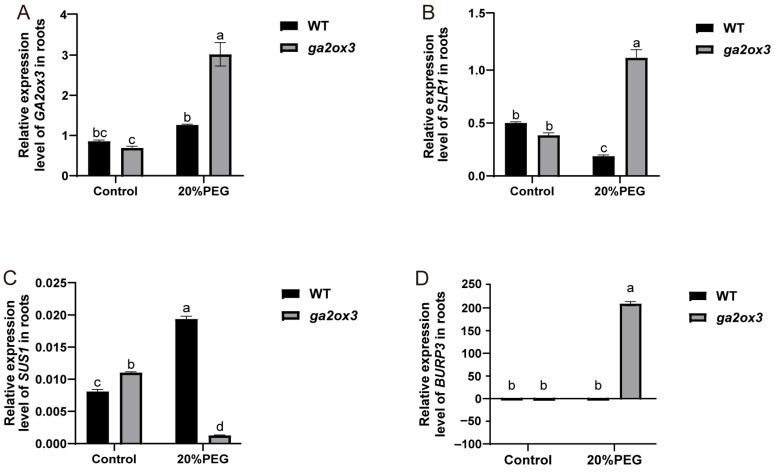
The expression of related genes in ZH11 and *ga2ox3* under normal conditions and 20% PEG conditions. The relative expression levels of (**A**) *GA2ox3*, (**B**) *SLR1*, (**C**) *SUS1*, and (**D**) *BURP3* in ZH11 and *ga2ox3* seedlings under normal conditions and 20% PEG treatment. Values are presented as mean ± SD from three biological replicates. The same letters indicate no significant difference between groups (*p* > 0.05), while different letters denote statistical significance (*p* < 0.05).

Under stress, *GA2ox3* expression was significantly upregulated by 1.5-fold in ZH11 and by 4.4-fold in *ga2ox3* compared to their respective controls (Figure 6A). Interestingly, *OsSLR1* and *OsBURP3* exhibited similar expression patterns after PEG treatment. In ZH11, their transcript levels decreased to 0.37-fold (*OsSLR1*) and 0.80-fold (*OsBURP3*) of control levels (Figure 6B,C). In contrast, in *ga2ox3* mutant, both genes were markedly induced, with *OsSLR1* increasing by 2.9-fold and *OsBURP3* by over 1200-fold relative to the control. Conversely, *OsSUS1* showed an opposite expression trend: it was strongly upregulated (23.8-fold) in ZH11 under stress, but dramatically downregulated (to 0.09-fold of control levels) in *ga2ox3* (Figure 6D). Together, these results suggest that the loss of *GA2ox3* function is associated with altered expression patterns of *OsSLR1*, *OsBURP3*, and *OsSUS1*, which may interfere with the GA-mediated transcriptional network and weaken the plant’s ability to respond to osmotic stress. The observed changes in expression among these components may contribute to the reduced tolerance to osmotic stress in *ga2ox3*.

### 2.6. GA2ox3^Hap1^ Enhances Drought Tolerance

**Figure 7:**
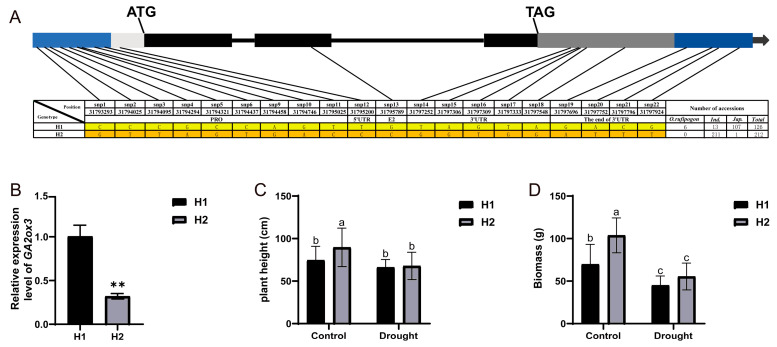
Effects of *GA2ox3* on plant height and biomass under drought stress in the field. (**A**) Schematic diagram of *GA2ox3* structure and haplotype analysis. Light blue rectangle represents the promoter region, white rectangle represents the 5′ UTR, black rectangles represent the exons, black lines represent the introns, gray rectangle represents the 3′ UTR, dark blue rectangle represents the end of the 3′ UTR. (**B**) The relative expression levels of *GA2ox3* in different haplotypes. (**C**) Plant height and (**D**) biomass of Hap1 and Hap2 under control and drought conditions. Values are presented as mean ± SD. Statistical significance was determined by Student’s *t*-test (** *p* < 0.01) The same letters indicate no significant difference between groups (*p* > 0.05), while different letters denote statistical significance (*p* < 0.05).

Resequencing of 627 rice accessions revealed 20 major SNPs within the *GA2ox3* genomic region (covering 2 kb upstream of the start codon, 5′ UTR, exons, 3′ UTR, and 1 kb downstream of the stop codon). These comprised nine SNPs in the promoter, one in the 5′ UTR, one in the second exon, five in the 3′ UTR, and four downstream of the stop codon (Figure 7A). Further analysis revealed that GA2ox3 could be classified into 109 distinct haplotypes (Hap1-Hap109) (Table S2), with Hap1 (reference sequence) and Hap2 being the predominant types.

Expression analysis in root tissues at 10 days after germination showed that the relative expression level of *GA2ox3* was significantly higher in genotypes harboring the Hap1 allele compared to those with Hap2. In addition, phenotypic evaluations were conducted under both normal and drought field conditions using rice varieties representing either Hap1 or Hap2 backgrounds. Under drought stress, the average plant height of Hap1-group and Hap2-group varieties was 66.0 cm and 69.07 cm, respectively, corresponding to reductions of 11.12% and 23.67% relative to normal conditions. Similarly, biomass decreased by 37.76% in Hap1-group and by 45.42% in the Hap2-group (Figure 7B–D).

Collectively, these results indicate that rice accessions carrying the *GA2ox3*^Hap1^ allele exhibit less reduction in plant height and biomass under drought stress, suggesting enhanced drought adaptability. This favorable haplotype may serve as a valuable genetic resource for molecular breeding aimed at improving drought tolerance in rice.

## 3. Discussion

Drought is one of the most devastating abiotic stresses affecting crop productivity, causing greater annual yield losses than the combined effects of all known pathogens [8]. With the intensification of global climate change, the frequency and severity of drought events are increasing, particularly in regions already prone to water scarcity. Over time, plants have evolved diverse strategies to cope with adverse environmental conditions such as drought. However, the underlying molecular mechanisms, especially those involving key genes relevant to drought adaptation and crop improvement, remain largely unresolved [16].

In this study, we investigated the role of *GA2ox3* in regulating drought stress responses in rice. Using CRISPR/Cas9-mediated gene editing, we generated *GA2ox3* knockout mutants (*ga2ox3*) in the ZH11 background. Phenotypic and physiological analyses showed that under normal conditions, loss of *GA2ox3* function resulted in increased plant height without affecting key yield-related traits. However, under 20% PEG-induced osmotic stress, *ga2ox3* exhibited more severe growth inhibition, excessive H_2_O_2_ accumulation, and reduced peroxidase (POD) activity compared to ZH11. While H_2_O_2_, MDA, and POD are commonly used markers for assessing oxidative stress, they represent only a subset of the complex antioxidant defense system. Further investigation into additional antioxidant enzymes and regulatory components would provide a more comprehensive understanding of the role of *GA2ox3* in oxidative stress responses. Gene expression analysis by qRT-PCR revealed that altered expression of *GA2ox3* was associated with changes in the expression of *OsSLR1*, *OsBURP3*, and *OsSUS1*, which may be related to the reduced tolerance to osmotic stress observed in the mutant lines; however, the underlying regulatory mechanisms require further experimental validation. In addition, haplotype analysis based on whole-genome resequencing of 627 rice accessions revealed that varieties carrying the *GA2ox3*^Hap1^ allele exhibited significantly higher gene expression levels and stronger drought tolerance than those with *GA2ox3*^Hap2^, suggesting that *GA2ox3*^Hap1^ represents a favorable natural allele for drought adaptation. Future studies could further incorporate more comprehensive physiological assessments (e.g., relative water content, chlorophyll content), validation using near-isogenic lines and transgenic materials, and in-depth dissection of upstream regulators and downstream target genes to elucidate the molecular regulatory network of *GA2ox3*-mediated drought tolerance.

In our study, plant height was significantly increased in *ga2ox3* mutants compared to the wild type, although these plants were still able to grow and develop normally, with no significant differences in yield. Consistently, Lo et al. reported that overexpression of *GA2ox3* led to severe dwarfism [15], and together these results indicate that *GA2ox3* negatively regulates plant height. These results suggest that different mutational disruptions within *GA2ox3* may have varying impacts on gene functions. Furthermore, consistent with results from Qin et al. [13], we observed no significant phenotypic differences between *ga2ox3* and the wild-type under normal conditions, despite the increase in plant height. However, our study additionally showed that *ga2ox3* mutants exhibited significantly shorter root length after PEG treatment, potentially reflecting reduced tolerance to osmotic stress. In the rice *GA2ox* family, different members exhibit distinct functional divergence in growth, development, and stress responses. For example, *OsGA2ox1* is primarily involved in the transition from vegetative to reproductive growth by regulating bioactive GA levels in the shoot apical meristem, affecting plant height and panicle development [17]; overexpression of *OsGA2ox5* causes extreme dwarfism and participates in gravitropism and salt stress responses [18]; different point mutations in *OsGA2ox6* confer enhanced dehydration tolerance, disease resistance, and yield, respectively [12,19]; knockout of *OsGA2ox7* improves seed germination ability under salt stress [20]; overexpression of *OsGA2ox8* enhances osmotic stress tolerance by increasing osmoregulatory substances and antioxidant enzyme activities [21]; and *OsGA2ox9* mainly regulates seed dormancy and pre-harvest sprouting [22]. Compared with these members, our study reveals that *GA2ox3* positively regulates osmotic stress tolerance, as its knockout leads to impaired antioxidant capacity and exacerbated oxidative damage, and this function is associated with the high-expression Hap1 haplotype in natural populations. This finding further enriches the functional diversity of the *GA2ox* family in stress responses. In maize, *ZmGA2ox1;1* is upregulated while *ZmGA2ox1;2* is downregulated under drought stress, displaying opposite regulatory patterns [10]. These findings collectively indicate that functional divergence and subfunctionalization are prevalent within the *GA2ox* family. Our study further supports this notion by demonstrating that natural allelic variation between *GA2ox3*^Hap1^ and *GA2ox3*^Hap2^ is associated with differences in gene expression and drought tolerance in rice, which aligns with previous reports.

Taken together, the identification of *GA2ox3* as a positive regulator of tolerance to osmotic stress provides valuable insights for rice genetic improvement. Although loss-of-function of other *GA2ox* family members has been shown to promote semi-dwarfism without compromising stress resilience in multiple crops [23], our study reveals that *GA2ox3* knockout in rice leads to increased plant height at the cost of osmotic stress tolerance, highlighting its role as a “growth–defense trade-off” gene. This trade-off is consistent with the established role of gibberellins in balancing growth and stress responses; for instance, DELLA proteins integrate environmental signals to modulate growth under adverse conditions [24], and manipulation of GA metabolism has been exploited to achieve semi-dwarfism without compromising stress resilience in multiple crops [23]. Such findings underscore the importance of fine-tuning GA homeostasis to optimize the trade-off between yield potential and stress adaptation. Notably, our haplotype analysis indicates that the *GA2ox3*^Hap1^ allele achieves a better balance by maintaining moderate gene expression-sufficient to suppress excess GA activity while preserving drought adaptability. These findings position *GA2ox3*, particularly the *GA2ox3*^Hap1^ variant, as a promising target for marker-assisted selection and genome editing in breeding drought-resilient rice cultivars, especially in regions frequently affected by water stress.

## 4. Materials and Methods

### 4.1. Plant Materials

The materials used in this study included the wild-type cultivar Zhonghua11 (ZH11) and two homozygous *GA2ox3* knockout mutants (*ga2ox3-1* and *ga2ox3-2*) generated in the ZH11 background using the CRISPR/Cas9 system. All plants were cultivated under natural field conditions at the experimental bases of Zhejiang A&F University in Lin’an, Hangzhou (30°18′ N, 119°43′ E) and Lingshui, Hainan (18°36′ N, 109°18′ E). The growing season was from May to October at the Lin’an site and from December to April at the Lingshui site. All plants were grown under natural light conditions.

### 4.2. Vector Construction and Generation of Transgenic Plants

The target site (5′-GTCGTCGACCTCGGCAGCCCCGG-3′) was designed in the first exon of *GA2ox3* using the online tool CRISPRdirect (https://crispr.dbcls.jp/) [25], and the knockout construct was generated using the pYLCRISPR/Cas9-MH/B vector [26]. Transgenic services were commissioned to Weimi Biotech Co., Ltd. (Changzhou, China), and Agrobacterium-mediated transformation of rice calli was performed. Regenerated plants were screened by Sanger sequencing, and two independent T0 homozygous lines were obtained, designated as *ga2ox3-1* and *ga2ox3-2*. Seeds from the T0 lines were harvested and propagated to generate T1 plants, which were used for subsequent phenotypic analyses. The target site (5′-GTCGTCGACCTCGGCAGCCCCGG-3′) was designed in the first exon of *GA2ox3* using the online tool CRISPRdirect [25], and the knockout construct was generated using the pYLCRISPR/Cas9-MH/B vector [26]. Transgenic services were commissioned to Weimi Biotech Co., Ltd., and Agrobacterium-mediated transformation of rice calli was performed. Regenerated plants were screened by Sanger sequencing, and two independent T0 homozygous lines were obtained, designated as *ga2ox3-1* and *ga2ox3-2*. Seeds from the T0 lines were harvested and propagated to generate T1 plants, which were used for subsequent phenotypic analyses.

### 4.3. Agronomic Trait Measurement and Data Analysis

Agronomic traits including plant height, tiller number, panicle length, number of grains per panicle, seed setting rate, and grain yield per plant were measured for both WT and *ga2ox3* lines. Statistical significance between groups was analyzed using Student’s *t*-test in GraphPad Prism 10.1.2. Differences with *p* < 0.05 were considered significant (*) and those with *p* < 0.01 were considered highly significant (**).

### 4.4. RNA Extraction and Gene Expression Analysis

Total RNA was extracted using Trizol reagent (Total RNA Extractor, Cat# B511311-0100, Sangon Biotech (Shanghai, China)) according to the manufacturer’s protocol. First-strand cDNA synthesis was performed using the HiScript III^st^ Strand cDNA Synthesis Kit (+gDNA wiper) (Cat# R312-02, Vazyme (Nanjing, China)), and the resulting cDNA was used for subsequent quantitative analysis. Quantitative real-time PCR (qPCR) was conducted on an ABI StepOne Plus Real-Time PCR System (Applied Biosystems (Shanghai, China)) using Taq Pro Universal SYBR qPCR Master Mix (Cat# Q712-02, Vazyme) to assess relative gene expression levels. Primer sequences used in this study are listed in Supplementary Table S1. The relative expression levels were calculated using the 2^−ΔΔCT^, with UBQ5 (*LOC_Os01g22490*) as the internal reference gene [14]. The primers for the relevant gene determination are all listed in Table S1.

### 4.5. Phenotypic Analysis Under Osmotic Stress

To investigate the physiological differences between ZH11 and *ga2ox3*, seeds were immersed in distilled water in a beaker and incubated at 37 °C in the dark for two days, followed by wrapping in a moist towel for one day to promote germination. Uniformly germinated seeds were transferred to 96-well plates and grown under controlled conditions (28 °C, 14 h light/10 h dark). Seedlings were successively cultivated in sterile distilled water for 2 days, half-strength Yoshida nutrient solution for 2 days, and full-strength Yoshida nutrient solution for 2 days, with the solution renewed every 2 days. On day 7, seedlings at the three-leaf stage were subjected to osmotic stress treatment by replacing the nutrient solution with 20% (*w*/*v*) PEG6000 dissolved in full-strength Yoshida nutrient solution. Control seedlings were maintained in full-strength Yoshida nutrient solution without PEG. The treatment lasted for 14 days, and the solution was renewed every 2 days to maintain consistent osmotic potential. At the end of the treatment period (day 14), phenotypic parameters including shoot length, root length, shoot fresh weight, and root fresh weight were measured.

### 4.6. Drought Treatment

The experiment was conducted from November 2021 to May 2022 at the experimental base of Zhejiang A&F University in Yazhou Bay, Hainan Province (18°36′ N, 109°18′ E). Rice seedlings were transplanted 25 days after sowing with a spacing of 20 × 20 cm, and each treatment was replicated three times. For the control group, normal irrigation was maintained after transplanting, whereas for the drought treatment group, no further irrigation was applied after transplanting until plant maturity. Phenotypic observations and statistical analyses were performed at the maturity stage.

### 4.7. Measurement of Physiological Parameters

To assess physiological differences between ZH11 and *ga2ox3*, several stress-responsive physiological indicators were measured, including hydrogen peroxide (H_2_O_2_) content, malondialdehyde (MDA) content, and peroxidase (POD) activity. After 20% PEG treatment, seedlings from both the control and 20% PEG groups were collected and ground in liquid nitrogen. H_2_O_2_ content (H_2_O_2_ Assay Kit, Cat# BC3595, Solarbio (Beijing, China)), MDA content (MDA Assay Kit, Cat# BC0025, Solarbio), and POD activity (POD Assay Kit, Cat# BC0095, Solarbio) were quantified using commercially available assay kits, following the manufacturer’s instructions.

### 4.8. Haplotype Analysis

Haplotype analysis of *GA2ox3* was conducted using whole-genome resequencing data from 627 rice accessions maintained in our laboratory. The analyzed region included 2 kb upstream of the gene, the entire coding sequence (CDS), and 1 kb downstream. Rare haplotypes present in fewer than 50 accessions were excluded from the analysis.

## 5. Conclusions

The present study investigates the positive regulatory role of *GA2ox3* in osmotic stress from a molecular functional perspective. Through the integration of haplotype analysis and phenotypic evaluation, a drought-tolerant superior haplotype, *GA2ox3*^Hap1^, was identified, providing candidate genetic resources for rice drought resistance breeding. These findings provide theoretical support and valuable genetic resources for improving stress resilience in rice, thereby contributing to agricultural adaptation to the challenges posed by climate change.

## Data Availability

The original contributions presented in this study are included in the article/Supplementary Material. Further inquiries can be directed to the corresponding author.

## Supplementary Materials

The following supporting information can be downloaded at: https://www.mdpi.com/article/10.3390/plants15081205/s1. Table S1: The list of gene-specific primers used for qRT-PCR; Table S2: Under normal field conditions and drought conditions, the data on plant height and biomass of various rice varieties.
