# Supplementary material for: Functional Characterization of GA2ox3 in Rice Osmotic Stress Response and Identification of a Superior Allele for Breeding Applications
Source: Plants (Basel). 2026 Apr 15;15(8):1205. doi: 10.3390/plants15081205 (PMC13119726; doi:10.3390/plants15081205)
Supplement: Supplementary file 1 [file plants-15-01205-s001.zip › plants-4216017-supplementary.pdf]

Table S1 The list of gene-specific primers used for qRT-PCR

Primer name	Gene locus	Primer sequence (5'-3')	Primer sequence (5'-3')
GA2ox3	Os01g55240	CCACCGTTAGCACAGAGGAT	CCTGTTGTCTCCAAGCCTTG
SLR1	Os03g49990	AGTCGCTGCACTACTACTCC	TACACCTCGGACATGACCTG
BURP3	Os01g53240	CAGCAACCTCCAGCTCAAGA	TGCTTGTTGCTGCTGCTCTT
SUS1	Os03g28330	CGGAAAGGGAATGCTTCAGG	CATCCTCAAAAGCACCGTCC
UBQ5	Os01g22490	CTCGCCGACTACAACATCCA	TCTTGGGCTTGGTGTACGTCTT

Table S2 Under normal field conditions and drought conditions, the data on plant height and biomass of various rice varieties

Variety	Haplotype	Plant height (cm)		Biomass (g)	
		Control	Drought	Control	Drought
QiMei1Hao	H106	70.775	66.98333333	74.675	47.965
YueT2567	H72	147.875	85.525	123.8	65.25
FengWei6Hao	H1	92.75		130.3333333	
XiaoBaiMang	H49	130.24	79.3	116.64	67.53
SanChaoQi	H106	136.74	95.08333333	126.78	68.745
LaoLongXu	H31	152.78	121.3583333	100.3	52.95
MuBangGu	H34	88.25		110.4333333	
HuoShaoHe	H43	89.92	80.68666667	41.05	48.14
HuangSiNuo2	H1	115.875		114.6	
ChaoAnGanYuan	H108	101.7	94.6	105.72	41
YiZuiHan	H2	138		200.4	
QiuBuLiao	H46	101.9	84.33333333	53.325	38.7875
ChenHaiBaiKeAi	H64	101.65	89.47	62.32	75.63
QiYuLengHe	H49	139	84.66666667	122.425	81.53333333
ZhongBaiHua	H106	110	92.1	115.5	49.88
NianKouHuang	H64	77.125	55.76666667		70.56666667
LongJing37	H1	55.775	56.23333333	72.18	54.09
HeiJing2	H1				
KenDao9	H1	75.8	70.54333333	93.475	34.98
KenDao88	H1				
DeLiQiEr90	H1	69.95	61.88	47.375	24.47
DeLiQiEr95	H1	60	65.36333333	30.6	20.8225
TaiZhong150	H1	86.78	82.16666667	83.926	45.915
LuHongZao1Hao	H53	52.8	55.65	53.36	52.18
TaiZhong145	H1	102	58.1	85.275	35.13333333
JiJing1Hao	H11	64.3	59.23333333	53.225	44.3975
HuNanZao1Hao	H2	58.625	59.81	67.18	55.75
XiNan175	H1	106.375	69.13333333	92.475	48.55
JinLing57	H86	132.75	65.25	97.16666667	41.6
JiaNan1Hao	H1	84.86	70.86666667	76.425	49.15
DongNong413	H11	64.35	68.16666667	37.44	43.37
Wen229	H51	83.375	63.26666667	68.625	66.2
XiangZaoXian1Hao	H9	80.5	59.76666667	108.6333333	53.5
SanErAi	H93	82.25	52.83333333	88.13333333	50.3
XianXiaoZhan	H97	80.75	61.1	61.76666667	62.8
TeXianZhan13	H2	111.75	65.23333333		61.76666667
QingSen5Hao	H1	71.075	74.9	54.05	49.105
Zhe76-1	H2	53.86	52.35833333	72.2	47.53

XianFeng1Hao	H2	60.36	59.1		53.8275
LianTangZao4Hao	H87	82.875		74.2	
XiangAiZao3Hao	H51	66.2	52.76666667	114.8	53.87
ChuanNong422	H64	83.2		125.325	
XiaoHongDao	H16	65.16	56.16666667		59.95
GuangJie9Hao	H53	92.75	75.96666667		46.06
GuangXuanZao	H2	65.08	56.03333333	93.84	
TuanJie1Hao	H99	68.1	60.75	111.72	51.855
Hong410	H88	70	67.6		56.66666667
SuDao1Hao	H1	88.15	77.2	113.8666667	39.65
SuDao2Hao	H13	68.72	62.21666667	68.2	60.94
WuNongZao	H1	69.5	68.08333333	68.34	46.27
ChengDuAi8Hao	H84	101.25		118.725	
HuShuang1011	H95	74.86	59.93333333	104.9	69.19333333
XinDao68-11	H11	73.46	70.2	51.74	47.7
JingYu1Hao	H1	70.1	73.7625	67.28	51.33
JingYue1Hao	H1	70.375	73.11666667	46.2	
GuiChao13	H67	73.42		103.88	60
ShuangGui1Hao	H83	80.875		82.725	
ShuangGui36	H2	106.325			
GuangEr104	H2	84.25	57.76666667	108.6	55.46666667
GuangErShi	H2	87.25	57.33333333	109.75	46.6
XiangWanXian1Hao	H2	62.4	68.73333333	76.35	44.1
ZiJinNuo	H1	55	61.46666667	52.95	58.39
SuZhouQing	H1	90.36	79.38333333	120.24	47.58
KunNongXuan16	H1	47.425	52.51666667	58.24	41.795
WuFuJing	H1	40.2	48.5	39.56	40.12666667
YangDao2Hao	H2	82.25	58.32666667	69.93333333	67.29666667
YanJing2Hao	H1	50.625	63.76666667	60.3	47.96
EZao6Hao	H2	74.74	60.53333333	112.25	75.575
ZhongHua8Hao	H2	65.875	65.05	76.05	43.94
HeJiang19	H1	55.9	63.65	30.075	35.27
Tong35	H2	66.5	66.75	101.48	49.2725
LiaoJing29	H1	72.125	80.08333333	64.3	67.21
SongLiao4Hao	H1	65.3	60.225	84.94	51.68
ZhongZuo93	H1	65.275	61.75	52	52.05
YuJing6Hao	H1	56.1	58.69666667	63.625	62.88
YuXian3Hao	H96	92.625		119.9666667	
LiaoJing454	H1	74.24	72.44	58.84	61.22
QiGuiZao25	H64	120.2	83.26666667	110.225	65
BeiHai1Hao	H1	75.15	80.53333333	38.5	48.13
GuoGuang	H1	96.58	80.7	143.025	63.6975
TaiNan5Hao	H1	111		106.8	
TaiNong70	H18	105		62	

AiZaiZhan	H1	58.975	54.24583333	86.6	50.3125
ZaoGui1Hao	H2	71.4	66.71666667	112.56	62.09
GanZaoXian51	H1	64.86	67.33333333	81.75	53.935
ZhongJian99-38	H96	69.4	67.31666667	116.5	53.88
HuaAi15	H50	63.54	59.63333333	96.425	53.16
WuZuiChuan	H1	97.02	71.00833333	206.2	49.17
MaFangXian	H71	124.92	84.8	196.64	44.8
ZheChang9Hao	H106	91	89.68333333	160.1	61.98
GuiHuaQiu	H1	79.6	71.2	95.125	44.27
LaoHuangGu	H66	109.5	78.21666667	145.7	76.06
YouZhan8Hao	H68	129.625		152.5333333	
SuXiangJing1Hao	H4	63.98	60.03666667	70.03333333	52.8
WuXiangJing14	H5	57.125	59.3925	70.36	47.43
EZao18	H86	77.45	70.56666667	149.6	70.31
WuJing15	H12	67.75	63.06666667	69.33333333	57.81
ZhenGuiAi1Hao	H55	79.075	58.98666667	126.55	59.81
XiangZao143	H93	62	60.87333333	81.78	66.28
JiaYu253	H91	65.4	70.58333333	75.08	64.24
LongJing04	H1	62.12	59.73333333	95.92	57.42
NanJing16	H90	67.75	57.86666667	91.4	51.33333333
WuYuJing5Hao	H1	56.75	57.66	60.88	53.4
WuYuJing9Hao	H1	55.96	60.3		64.4
YangFuXian6Hao	H49	91.75		69.76666667	
ZhongZao35	H2	70.925	68.83333333	89.82	110.8
ZhongZao39	H2	66.225	69.425	76.48	52.645
SuXiu10Hao	H1	54.76	58.7375	50.525	52.52
XiuShui123	H1	56.65	54.5	65.98	60.96
Tadukan	H100	148.625		135.2	
Bei Khe	H106	103.16	90.22	131.88	47.27
Jena035	H64	156.75	117.1	52.06666667	78.58
Naba	H64	115.1666667			
Davao1	H96	134.25		117.2666667	
Ryou Suisan Koumai	H2	113.25	100.34	90	41.17
Jinguoyin	H64	130.52	98.07083333		85.57
Dahonggu	H64	143	110.375	143.275	86.78
Asu	H64	112.82	67.46666667	151.3333333	27.1
Co13	H106	102.64	85.58333333	143.1666667	55.14
Vary Futsi	H2	116.75	94.1	81.73333333	42.06666667
Keiboba	H2	132.9	80.65		50.97
Qingu(Seiyu)	H76	143		104.65	
Deng Paozhai	H96	143.5		143.675	
Shwe Nang Gyi	H2	115.375		76.4	
Calotoc	H60	113.25		71.775	
Lebed	H2	155		115.6	

Pinulupot-1	H2	140.125		112.0666667	
Muha	H64	111.14	80.31666667	124.48	52.015
Jhona2	H70	95.3	82.07		43.5325
Nepal8	H64	79.83333333	76.4	58.83333333	60.23333333
Jarjan	H64	147.625	117.76	109.6	92.62
Kalo Dhan	H64	138.42	127.4	92.64	63.65
Anjana Dhan	H64	119.65	79.88333333	143.925	57.76
Shoni	H63	93.875	78.03333333	49.6	49.3
Tupa 121-3	H63	128.875	77.03333333	119.475	53.66
Surjamukhi	H62	124.375	76.23333333	90.2	50.97333333
Arc7291	H64	106.12	86.81666667	74.6	46.86
Arc5955	H64				
Ratul	H62	117.875		91.18	
Arc7047	H64	126		82.25	
Arc11094	H61	124.625	86.6	87.575	72.54
Badari Dhan	H63		65.75		32.64
Nepal555	H62			165.45	
Kaluheenati	H62	82.175	78.81	111.94	33.93
Local Basmati	H60	97			
Dianyu1	H1	118.5	99.7	101.8333333	
Basilanon	H2	138.3333333		143.875	
Ma Sho	H14	87.06	69.5	100.9	46.1
Khao Nok	H34	118.75			
Jaguary	H102	115.36	84.41666667	136.36	70.35333333
Khau Mac Kho	H34	135.625	85.75	104.675	52.92
Padi Perak	H34	94.25		109.7	
Rexmont	H34	74	62.38333333	55.4	37.20333333
Khau Tan Chiem	H34	144.125		115.45	
Tima	H34	129.7	121.5333333	62.7	36.35
Tupa729	H69	127.05		147.78	
Hong Cheuh Zai	H81	132.4		158.8	
Urasan 1	H48	112.825		118.98	
Bingala	H35	102.5		97.25	
Vandaran	H2	103.92		101.95	
NiuMaoHuang	H17	77.84	79.7	69.54	57.37
ErJiuFeng	H98	57	62.26666667	57.6	54.5525
ShuangNuo4Hao	H8				
XiangHu25	H1				
Zhong156	H83	61.525	61.43333333	53.54	54.04
Ning67	H9	56.425	57.6	55.32	48.88
Zhong86-44	H54	136	58.83333333	125.8	55.3475
ZhongSi2Hao	H1	60.4	56.21666667	74.02	46.58333333
JiaYu293	H92	52.7	58.25	48.9	52.71

ZhongZao4Hao	H49	57.5	66.66666667	84.98	73.55
NongHong73	H1	69.125			
NongHu3-2	H1	71.9	66.23333333	123.26	52.465
AiNanZao1Hao	H47	46.23333333	52.61666667		47.85
XiuShui48	H1	68.56	63.1	103.175	42.2375
AiJiaoNanTe	H96	69	56.37	97.875	58.2975
ChangBai6Hao	H1	68.025	71.56666667	63.3	38.185
YuChi231-8	H2	67.2	58.03333333	107.8	15.4
ZhongDan2Hao	H1	93.4	87.4	125.74	55.33
IR22	H91	58.475	58.95	33.46	33.91
IR29	H91	78.125		78.025	
GaoXiong10Hao	H1	112.75		78.075	
GaoXiong18Hao	H1	96.66666667		61.16666667	
JiaNan22Hao	H1	111.6666667		87.575	
GaoXiong22Hao	H1	115.875		86.875	
ZhuXi26	H87	63.2	56.9	62.3	62.12
ZhongZao22	H52	68.275	65.85	75.6	39.9125
WuYunJing8Hao	H11	59.28	55.33333333	60.28	58.24
YueXiangZhan	H3	66.6	62.71666667	84.8	49.8
YinFang	H92				
N6700	H31				
N3110	H33				
N5640	H42				
N7401	H23				
2231	H1				
N6151	H41				
N391	H29				
3442	H1				
2742	H101				
3252	H44				
852	H32				
3182	H7				
1382	H1				
3234	H74				
144	H25				
444	H31				
364	H31				
N6205	H22				
2045	H23				
345	H23				
N1955	H29				
485	H27				
195	H24				
N295	H24				

46	H28				
1056	H31				
956	H3				
N3476	H73				
7	H24				
27	H23				
1827	H15				
1947	H36				
657	H1				
1167	H21				
897	H30				
N7218	H26				
2188	H1				
429	H26				
2729	H10				
3349	H1				
3449	H31				
2549	H31				
FFZ1	H49				
PR 33282-B-8-1-1-1-1-1	H1				
IR 02A127	H106				
IR 68	H2				
SAGC-08	H2				
QingLian16	H106	64.52	58.91666667	41.2	41.34
BP1976B-2-3-7-TB-1-1	H80				
CT 16658-5-2-2SR-2-3-6MP	H106				
XinChangJing	H1				
TianFeng	H2	59.88	52.6	66.44	38.5875
ReYan	H34	78.65	67.38333333	75.875	31.80833333
Kutube-n	H34	138	70.66666667	105.8333333	47.33333333
Gamilunan	H34	134.625	90.86666667	100.775	32.64166667
Radi Pagalou	H60	139.125		131.525	
Chabli chabol Glang	H34	95.225	82.43333333	41.4	47.25
Kenteng	H34	118.875	103.4833333	55.23333333	31.51666667
Kasinkan	H60				
IRAT 243	H34	115.125	74.66666667	72.575	25.94166667
SELECCION 34-1B	H1	78.16666667	58.3	94.53333333	30.5
GaoLiangDao	H1				
NanYangZhan	H34	131.5		112.725	
Albolia Plecoce	H89	89.5	64.3	158.5666667	62.53333333
Arborio	H106	107.98	78.98333333	80.55	42.18666667
SLG-1	H34	97.75	80.66666667	69.53333333	35.86666667
Hrborio cyauco	H1		66.06666667	39.4	52.8575
Baldo	H1	78.22	81	51.85	48.85

IRAT 104	H34				
AErBaNiYa	H1	69.1	65.68	29.15	65.68
JiaNaDa1Hao	H75	51.7	56.68333333	55.15	49.14
LanDao	H1	111.5	76.88333333	85.575	52.675
AiZiHuangKeNuo	H1				
AoYu326	H1	71.36	67.26666667	49.56	45.34666667
SuYuNuo	H2	120		99.26666667	
SanLiCun	H34	102.68	101.5866667	48.66	47.28
TaiGuoXiangDao	H2	58.2	58.98	48.44	59.33
GeLuo27	H34	103.625	91.41666667	84	78.75833333
RD25	H96		78.58333333		60.56666667
Barah	H60	95.05	85.88333333	55.525	77.55
DR32	H2	79		123.2333333	
C.MEDIO 18	H94	74.75	65	107.275	80.7
CEYSVONI SML	H34	95.45	69.13333333		60.9
BeiLu129	H1	77.74	68.3	72.08	62.29333333
ECIA 66	H2	81.75		112.9666667	
PANAMA 1537	H2	81	58.8	112.15	47.16666667
C91 CU81-SM1NA	H96	67	53.21666667	90	51.88
ITA 233	H1	98.5		53.86666667	37.63333333
63-83(Acc.14725	H34	120			
C8CU80-SMCU-3CU-1NA-2CU-1CU	H2	58.35	55	86.3	42.19333333
MiYang46	H2	68.25		89.24	
ShuHui527	H1	96.5		77.36666667	
BianBie	H31	82.8	82.85	52.26	51.675
RenHuaJiuGongJi	H2	85.8	75.33333333	57	37.21
ChuanDaLi	H106	89.275	76.3	87.45	80.2
ZaoShuChangKeGangNuo	H34	109.74	98.68333333	54.725	28.40333333
Zhong242	H34	112		94.45	
TeDaZhan	H34	115.875		125.4666667	
BaoDaLi	H75	116.1			
QiGuiB	H2	87.375	71.23333333	48.275	54.23333333
KuangSiJiaDi	H2	71.33333333	61.2	75.75	40.8
LuoChangDaLi	H106				
LuoDaSui	H1				
XiuShui09	H1	77.925	55.65	42.8	51.75
Zhi4	H2	83.125	56.5	112.0666667	57.76666667
TaiZhong65	H6				
XinChangJing	H1	87.75		92.85	
TaiHuQing	H4		73.31666667	49.72	54.0925
GaoLiangDao	H1	53.2	58.7	30.1	56.09
DaLiGuaNuo	H26	74.1	89.06666667	34.6	37.78
WanBaiGe	H4	80.72	75.44	49.8	46.2875

FengZiNuo	H26	100.66	86.11666667	69.92	45.19
AiZiHuangKeNuo	H1	119	98.56666667	66.65	55.31666667
DaoHuaXiang2Hao	H1	91.6	90.75	82.36	42.31333333
ShengLiXian	H64	123.125	94.13333333		69.25
XiangZaoXian13Hao	H93	85		64.9	
JiaYu948	H79	60.875	62.26	93.275	60.025
YangFuXIan5Hao	H49	98.375			
IR36	H99				
YingDeDaYePai	H77	136.625	67.76666667	122.7333333	61.33333333
EWan5Hao	H1				
WuYunJing7Hao	H1				
XuDao3Hao	H1				
XiuShui09	H1				
XinZhu4Hao	H1				
NongHu6Hao	H1				
HeJiang23	H1				
QiuGuang	H1				
XiangHu84	H1				
WuYuJing3Hao	H1				
DongNong416	H1				
ChangBai9Hao	H1				
XiuShui63	H11	66.64	60.33333333	50.88	73.3
ZaoFeng9Hao	H1				
KenDao8Hao	H49	99.225	85.68333333	148.15	71.8775
KongYu131	H1	45.275	58.9	19.15	42.38
EYi105	H12	73.72	66.3	87.125	62.85
HuNanZao	H2	134.25	87.73333333	202	78.46666667
XiangZaoXian3Hao	H49	58.225	53.45333333	67.56	36.2975
Zhe733	H2	65.36	57.41666667	92.68	39.925
ZheFu802	H2	70.275	49.9		27.85
WanLiXian	H64	131.34			
LuCaiHao	H49	95.4	91.91666667		54.21666667
ZhwChang3Hao	H106	128.28	87.21666667	81.32	38.3
ZhaiYeQing8Hao	H107	73.3		113.5	
YuanFengZao	H96	63.075	62.43333333	75.93333333	55.63
GuiChao2Hao	H2	108.375		217.525	
XiangZaoXian7Hao	H64	101.84	83.25	84.05	45.94
JingXian89	H2	76.3	55.13333333	105.25	36.8
Zhou903	H85	72.25	65.9	72.725	57.47666667
GanZaoXian37	H1	64	67.7		59.35
GuangLuAi4Hao	H2	56.16	51.75	86.15	47.89
XiangAiZao9Hao	H49	63.725	58.2	92.96	71.27666667
ErJiuQing	H2	60.425	55.13333333	44.54	56.94
HuangZhan(5)	H64	69.55	72.36666667	77.26	53.775

XiaoMaoXiang	H64	132.75	82.56666667	193	55.96666667
YangJianNuo	H45	112.28	71.08333333	83.8	50.825
HuaZhan	H106	111.76	77.43333333	98.9	46.13666667
RuanWuDao	H1	78.9	72.35	62.7	27.35
QingZhong	H106		61.01666667	36.475	38.08
DaLiHong	H64	89.42	79.95	39.8	37.94
HeiZhuNiao	H1				
LiuTiaoZi	H64	100.42	80.28333333	66.6	45.175
Ichsannde	H64	138.5		146.5	
IFON	H64	143.5	88.5	131.3	75.05333333
488	H1	73.975	66.23333333	66.375	50.02666667
Labelle	H34	104.75	92.3	139.925	41.65
JinBaoYin	H106				
YunJing23Hao	H2				
Basmati 385	H1	91.275	77.76666667	90.4	44.1
FanHaoPi	H64	108.62	89.13333333	97.83333333	46.06666667
MaGuZi	H40	92.94	79.91666667	65.025	36.98666667
HanMaDao	H103	110.075	75.4	88.4	38.56666667
ZhongNong4Hao	H2	128.5	75.63333333	153.2333333	79.93333333
GuMei2Hao	H2	111.2		59.925	
MaMaGu	H64	108.1	92.9	99.96666667	39.4
IR64	H2	111.05		173.9333333	108.8333333
Tetep	H76	61.95	58.76666667	33.33333333	23.5575
GuiHuaHuang	H1	54.275	56.2	46.48	41.44
CPSLO17	H34	81.725	69.98333333		58.00833333
GuanDong146	H1	48.4	55.06666667	30.72	32.77
TN1	H2	57.275	54.28	58.28	42.45
SanLiCun	H1	110.25		96.13333333	
MiYang46	H2				
KunMingXiaoBaiGu	H37	118.075	100.0833333	63.88	24.225
LiJiangXinTuanHeiGu	H31	55.075	49.35	54.92	24.24
HuaZhan	H2	77.25		183.95	145.9
WuShanYouZhan	H105	77.375		120.725	
WuYunJing3Hao	H1	43.66	44.10833333	51.2	29.14333333
lemont	H34	73.5	62.43333333	67.7	39.1
Rethu Heenati	H106	70.325	56.93333333	51.275	24.66666667
SWR17	H17				
Cypress	H14	71.5		113.5333333	
Tianfeng（Pigm+Xa23)	H2	83.66		126.16	
XiuShui11	H1				
TianFeng	H96				
XiXuan4Hao	H2	62.32	60.08333333	77.82	41.26
HuangHuaZhan	H2	77.75		133.725	115.9
SiMiao	H106	80.2	76.45833333	59.98	40.4125

LongGe113	H2	58.1	47.5	67.22	25.825
BaiKeZaoHe	H49	99.15		129.125	
YiZhiXiang	H106		107.2666667	92.14	42.85
GuangDaBai	H2				
XuanEnChangTanQingZhan	H65	106.3	85.98333333	68.56	49.63
NanBaoZao	H2	66.68	58.75	99.44	44.61
MinKeZao1hao	H1				
ZhenMi85	H78				
DiJiaoWuJian	H49	59.8	53.6		41.65
9311	H59	88.625		173.4	
HuangKeZaoNianRui	H20	75.28	75.11666667	60.275	42.7825
HaoBaYong1	H34	86.54	89.53333333	57.025	31.85666667
77-175	H19	65.925	53.11666667	93.375	44.12333333
HongYun33	H106	86		175.45	
JinNanWan	H2	68.875		125.2	
IIXiang	H2	74.6	59.85	95.375	49.40666667
YueFengB	H2	72.7	57.36666667	114.8	31.33333333
XieQingZaoB	H2	58.375	53.7	89.3	40.2475
HuaJingXian74	H2	78.125	61.66666667	159.45	39.13333333
IR58025	H2				
PeiAi64	H49	57.825	50.4	89.18	60.73333333
IR36	H2				
IR32	H2	93.6	59.6	129.18	84.28
YueGuang	H1	51.2	42.29	53.9	26.18
IR26	H106				
ChunJiang06	H2	56.8	49.7	59.22	27.67666667
Gui630	H2	72.5		107.7	
DuoXi1Hao	H2	81		132.475	
BoBai	H2	59.96	53.91666667	58.5	40.95666667
LongTePu	H1	67.8		84.84	
Pusa33	H104				
IR8	H2				
IR56	H2	70.98	62.9	73.975	32.35
YunJing37	H1	77.925	71.4	79.04	40.01333333
YanFeng47	H1	62.125	62.8		35.73
WuYunJing7Hao	H1	65.54	67.58333333	49.05	35.77666667
Tian4Nuo	H76	91.75		174.925	
NanJing46	H1	32.1	46.08333333	14.04	29.27
Peta	H2				
MiYang42	H76	72.75		48.325	
GuangDong51	H1	80.7	68.43333333	99.375	35.7
IR24	H2	95.35	79.76666667	117.4	101.2833333
ZheFu802	H2				
LongJing21	H1	65.2	68.9	35.3	31.74

LongJing46	H1	47.2	62.7		37.37
SuiJing4Hao	H1				
CBao	H1				
LanSheng	H1				
HeXi22	H31	99.25		115.2666667	
HaoBuKa	H64	130.75		157.0666667	
ChuJing27	H1	80.75	63.8	57.075	36.75
GanXiang	H106	67.32	69.9	72.9	26.9
NeiXiang7	H31	64.6		48.075	
GuangLuAi4Hao	H2				
Wanlox	H1	59.825	58.13333333	74.125	37.43
Lenon	H34	63.72		45.18	
JiaHe212	H1	47.85	59.88333333		26.07083333
BeiTianDao	H2	78.18		128.1	
ZhongZao11	H2	64.86	57.41	67.7	43.8275
ZhanGu	H2	55.45	56.76666667	82.24	27.05
IR 40	H2				
Daw Dam	H39	84.575	78.45833333		31.44
Dahanala 2220	H61	89.72	65.5		36.6
Asominori	H1	63.8	61.65		33.06
ZhenShan97	H1	65.8	51.08333333	89.54	32.65333333
ZaoXianDang	H49	59.275		101	
AiLiShu	H1	60.52	58.4	50.4	40.56
DongYe1Hao	H1				
R308	H2	67.625	58.96666667	119.75	60.815
TaiDongLuDao328	H60	74.3	89.63333333	55.175	53.92333333
HuangSiGuiZhan	H49	59.75	54.5	67.42	49.24
BaWangBian1	H2	68.78	83.00833333	76.7	43.73333333
ZaoShuNongHu6HaoB	H1	58.1	57.23333333	49.24	42.23
XianGaiB	H2	68.33333333	51.63333333	80.36666667	59.23333333
C418	H1	86.68	69.41666667	75.52	30.07
MingHui63	H2	81		136.55	
NSIC-RC150	H2				
NSIC-RC152	H2				
NSIC-RC154	H2				
NSIC-RC160	H2				
NSIC-RC216	H2				
RC224	H2				
RC226	H2				
PSB-RC32	H2				
PSB-RC58	H2				
NSIC-RC156	H2				
IR24	H2				
RC240	H2				

RC308	H2				
RC298	H2				
RC300	H2				
RC354	H2				
IR26	H2	109.7		92.175	
IR32	H2				
IR36	H2	109.225	52.86666667	170.92	64.74
IR42	H2	102.3		87.95	
IR50	H2	60.23333333	51.83333333	121.18	46.21666667
IR58	H2	57.45	47.76666667	89	38.28
IR60	H2	84.3	79.25	85.1	54.425
IR62	H2	71.875			
IR64	H2				
AS994	H2				
IR65600-81-5-3-2	H1				
IR66	H2	68.25	63.1	143.6	68.01666667
QianLiDao	H56				
Jamaica	H38				
BaLiLa	H1				
kasalath	H64				
BaoDaLi	H2				
KF1	H2				
ChaunYou6203	H76				
IR70	H2	81.5		100.675	80.72
YiXiang215	H109				
N411	H34				
Yao	H76				
TaiZhan	H2				
PGF-1	H2				
IR71700-247-1-1-2	H2				
IR72	H2	64.75	54.93333333	53.88	45.61333333
IR74	H2				
IR8	H2				
IRRI102	H2				
IRRI103	H2				
IRRI104	H2				
BR29	H2				
IRRI105	H2				
IRRI106	H2				
IRRI108	H2				
IRRI109	H76				
IRRI115	H2				
IRRI116	H2				
IRRI118	H2				

IRRI119	H2				
IRRI122	H2				
IRRI123	H2				
IRRI134	H58				
IRRI135	H57				
IRRI136	H2				
IRRI141	H2				
IRRI145	H2				
IRRI146	H2				
IRRI147	H2				
IRRI148	H2				
IRRI150	H2				
IRRI151	H2				
CIGEULIS	H2	108.65	47.33333333	130.14	99.2
IRRI154	H2				
IRRI156	H2				
IRRI161	H2				
IRRI162	H2				
NSIC2013	H2				
IRRI168	H2				
IRRI172	H2				
IRRI174	H2				
IRRI179	H2				
CIHERANG-SUBI	H2				
IRRI181	H2				
Jasmine 85	H2	79.125		85.825	
Manawthukha	H106	95.125		112.06	
Mekongga	H76	83.375			
Milyang23	H2	99.125		94.72	
MTU110	H2				
NSIC2011	H2				
RC240	H2				
OM4900	H76				
OM5629	H1				
INPARI26	H2				
OM6161	H2				
OM6162	H2				
NSIC-RC141	H2				
OM6600	H2				
OM6677	H2				
Sabitri	H2	71.5		88.8	
Saltolsinthwelatt	H106	0			
Sambamahsun	H2	65			89.52
Sanbamahsun-Subl	H106				

INPARI27	H2				
PSB-RC74	H2				
TDK 11	H2				
TDK 8	H2				
TDKL-Subl	H2	84.625		170.675	
IR5564-44-2-3	H2				
IR72158-16-3-3	H2				
IR73707-45-3-2-3	H2				
BR11	H2				
Cisadane	H109	92.625		199.8	88.275
Mahsun	H61	86.9	52.55	127.38	48.68666667
INPARI28	H2				
PSB-RC6	H2				
PSB-RC4	H2				
PSB-RC8	H2				
NSIC120	H2				
NSIC128	H2				
NSIC130	H2				
NSIC134	H2				
NSIC138	H2				
NSIC142	H2				
NSIC146	H2				
IR20	H2	100.2	56.05	92.7	35.73333333
BHURARATA4-10	H82				
POKKALI	H2				
NONABOKRA	H2				
POKKALI(8558)	H2				
FL478	H2				
POKKALI::IRGC 108921-C1	H2				
IR 66946-3R-178-1-1	H2				
NSICRC106	H2				
CHERIVIRUPPU	H2				
WALIMBO	H64				
CSR28	H2				
TCP-266-2-49-B-B-3	H2				
IR 51491-AC 10	H2				
BPIRI 2	H106				
ARC18567	H2				
MSALIMJARO	H2				
POKKALI 4	H2				
JUMBOJET	H106				
IR8866-30-3-1-4-2	H2				
IR45427-2B-2-2B-1-1::G1	H2				

ORUMUNDAKAN	H64				
GUNDANG	H106				
SOM	H106				
QingXiaoJinZao	H60	85.125	56.86666667		50.2
TIMBOU	H60	115.6666667		117.3666667	
PSBRC 50	H2				
GETU	H2				
TAL MUGUR	H64				
AKUNDI	H2				
QingHuaAi6Hao	H64	65.7	51.75	109.4	43.33
RD 23	H2				
BRRI DHAN 53	H106				
CSR 13	H2				
RAYADA	H34				
ShuHui202	H60				
PATNAI	H2				
TA LAY	H64				
KUATIK PUTIH	H106				
CSR 11	H2				
ZF802	H96				
